# Case Report: New phenotype of late-onset Stüve–Wiedemann syndrome due to a C-terminal variant in the *LIFR* gene

**DOI:** 10.3389/fped.2024.1442624

**Published:** 2024-10-31

**Authors:** Evgenia Melnik, Margarita Sharova, Vladimir Kenis, Anna Morgul, Viktoria Zabnenkova, Tatiana Markova

**Affiliations:** ^1^ Research and Counseling Department, Research Centre for Medical Genetics, Moscow, Russia; ^2^ H. Turner National Medical Research Center for Children’s Orthopedics and Trauma Surgery of the Ministry of Health of the Russian Federation, Saint Petersburg, Russia; ^3^ Laboratory of Molecular Genetic Diagnosis 3, Research Centre for Medical Genetics, Moscow, Russia

**Keywords:** Stüve–Wiedemann syndrome, new phenotype, *LIFR*, arthrogryposis-like phenotype, C-terminal variants

## Abstract

An early diagnosis of Stüve–Wiedemann syndrome is crucial due to its high neonatal lethality and the potential for autonomic dysfunction in children. Herein, we describe a patient with a late-onset, arthrogryposis-like phenotype form of Stüve–Wiedemann syndrome. While most cases result in neonatal complications, our patient only presented with camptodactyly, ulnar deviation of the wrist, and minor facial features at birth, resembling an arthrogryposis-like phenotype. The condition went undiagnosed until adolescence when noticeable gait and posture abnormalities emerged. Clinical and radiological findings confirmed the diagnosis of benign Stüve–Wiedemann syndrome with light autonomic dysregulation. Notably, our patient lacked the typical bent bone features but showed widened metaphyses and thickened femoral necks. Genetic analysis revealed a novel variant in the last exon of the *LIFR* gene, possibly explaining the mild phenotype. This case expands our understanding of Stüve–Wiedemann syndrome variability, aiding in earlier detection and better medical-genetic counseling.

## 1 Introduction

Stüve–Wiedemann syndrome [SWS; Online Mendelian Inheritance in Man (OMIM):601559] is an ultra-rare autosomal recessive skeletal dysplasia from the group of “bent bone dysplasia” syndromes and is also associated with dysregulation of the autonomic nervous system (1, 2). It can be suspected during prenatal ultrasound examination if intrauterine growth retardation, shortening and bending of the long bones, camptodactyly, oligohydramnios, and fetal hypokinesia are found (3) In addition, specific radiological signs of SWS include widened metaphyses with abnormal trabecular patterns and a thickening of the cortical layer of the concave part of the bent long bones (1, 4).

Stüve and Wiedemann first described the disease in 1971 in two siblings, both of whom died shortly after birth (5). Historically, SWS was considered lethal in the neonatal period or within the first 2 years of life due to the development of neonatal respiratory distress syndrome, dysphagia, and life-threatening episodes of malignant hyperthermia. However, in recent years, there has been an increasing number of reports of patients in older childhood (6, 7). It has been shown that the SWS in infant survivors is characterized by progressive lower limb deformities, increasing joint contractures, severe scoliosis, and short stature (6, 8, 9). Moreover, long-term complications include osteopenia and pathological bone fractures (4, 10). Despite the normalization of breathing and swallowing in survivors beyond the first year, autonomic nervous system dysregulation symptoms in the form of thermoregulatory disturbances and paradoxical sweating are experienced in 88% of cases, often accompanied by decreased pain sensitivity, absence of a corneal reflex, and a smooth tongue (6). Furthermore, the majority of patients exhibit enamel defects, chronic abscesses, and early tooth loss (7, 11).

The association between the *LIFR* gene and the syndrome was first established in 2004 by Dagoneau et al., who first identified null variants in the *LIFR* gene in 19 families with SWS (1). More recently, homozygous variants in the *IL6ST* gene have been found in some patients with lethal SWS, confirming its heterogeneity (SWS, type 2; OMIM: 619751) (12). The *LIFR* gene encodes a receptor that binds to certain cytokines from the interleukin-6 (IL-6) family that initiate the JAK/STAT3 signaling pathway, including leukemia inhibitory factor (lif) (1, 13). Protein complexes within this signaling pathway stimulate the proliferation and differentiation of osteoblasts and osteoclasts and influence the cholinergic differentiation of the sympathetic nervous system, myoblast differentiation, and muscle tissue growth (1).

Currently, 35 pathogenic variants in the *LIFR* gene have been described in patients with SWS according to the Human Gene Mutation Database (HGMD® Professional 2022. 1) and these are typically loss of function variants. Clear genotype–phenotype correlations have not been established to date. However, there are some reports on the clinical heterogeneity of SWS, including incomplete phenotypes (7, 14, 15). Taken together, this necessitates the study of the clinical manifestations of rare non-lethal cases of SWS caused by newly identified variants in the *LIFR* gene.

Herein, we present the case of a new phenotype of SWS caused by a novel variant in the C-terminal domain and previously described variants in the *LIFR* gene in a compound heterozygous state and provide an analysis of the clinical and radiological characteristics of the 15-year-old patient.

## 2 Materials and methods

A comprehensive examination of the 15-year-old male proband was conducted, who exhibited features of skeletal dysplasia combined with an arthrogryposis-like syndrome and manifestations of autonomic dysfunction. To refine the diagnosis, clinical-genetic analysis; electromyography (EMG; surface and needle); x-rays of the spine, hip joints, and long bones of the extremities; and molecular genetic testing were utilized.

Written informed consent was obtained from the proband's parents. The study was performed in accordance with the Declaration of Helsinki and approved by the Institutional Review Board of the Research Centre for Medical Genetics, Moscow, Russia.

Blood samples were collected from the proband and his unaffected parents, and genomic DNA was extracted by standard methods using a Wizard Genomic DNA Purification Kit (Promega, WI, USA). Clinical exome sequencing was performed to identify mutations in the proband’s DNA. Target enrichment using a SeqCap EZ HyperCap Workflow solution capture array (Roche Sequencing Solutions, CA, USA) included the coding regions of 6,640 genes currently described as clinically significant in the OMIM and HGMD. Paired-end sequencing (2 × 75bp) was carried out on an Illumina NextSeq 500. Sequencing data were processed using a standard computer-based algorithm in Illumina BaseSpace software (Enrichment 3.1.0). Mapped reads were visualized using the Integrative Genomics Viewer (IGV) software (© 2013–2018 Broad Institute, MA, USA, and University of California, San Diego, CA, USA). The variant filtering algorithm was based on a frequency of less than 1% in The Genome Aggregation Database (gnomAD v.2.1.1) and coding region sequence effects such as missense, nonsense, coding indels, and splice sites. The clinical significance of the variants was evaluated according to the massive parallel sequencing (MPS) data interpretation guidelines (16). The cDNA and protein positions in *LIFR* corresponded to transcript NM_002310.6. Identified variants in the *LIFR* gene were validated using Sanger sequencing by standard protocols using a 3500xL Genetic Analyzer (Applied Biosystems, Thermo Fischer Scientific, MA, USA).

## 3 Results

### 3.1 Clinical findings

The proband, a 15-year-old boy, was examined due to complaints of altered gait; finger joint deformations; restricted movement in the interphalangeal, radiocarpal, and elbow joints; back muscle weakness; and spinal curvature. The child's parents were healthy, not consanguineous, and had a younger healthy daughter. The mother's first pregnancy ended in a miscarriage at 13 weeks. The proband was born in her normal second pregnancy with a birth weight of 3,990 g [0.93 standard deviation score (SDS)], a length of 52 cm (0.70 SDS), and an APGAR score of 8/9. Camptodactyly and ulnar deviation of the wrists were observed from birth. Congenital arthrogryposis was suspected. Facial phenotypic features were noted, including a prominent forehead, mild hypomimia, and microstomia. During his first year of life, the child experienced feeding difficulties due to a weak sucking reflex and episodes of choking during feeding, leading to a weight deficit. Early motor development occurred with slight delays: head control at 5 months, supine rolling at 5 months, sitting at 10 months, and walking at 1 year and 3 months. Spontaneous bone fractures in his forearms and lower third of the shin began at the age of 5 years (one to two times a year), with the last fracture of the left radius occurring at 11 years of age. Restriction of movement in the small and large joints of his upper extremities developed from the same age.

Furthermore, at the age of 11, his parents noticed that he overheated quickly and could not tolerate hot weather. Hypohidrosis and sudden facial flushing were also noted. Hypohidrosis turned to paradoxical episodes of profuse sweating that required the changing of clothes five to six times a day, even in winter.

At the examination of the patient at the age of 15, his height was 181 cm (+1.38 SDS) and his weight was 63 kg (+0.42 SDS). The patient had a high forehead, epicanthal folds, narrow palpebral fissures, a short deep philtrum, a smooth nasolabial triangle, mild hypomimia, and a high palate. Follicular keratosis-like eruptions were observed on his forearms.

**Figure 1:**
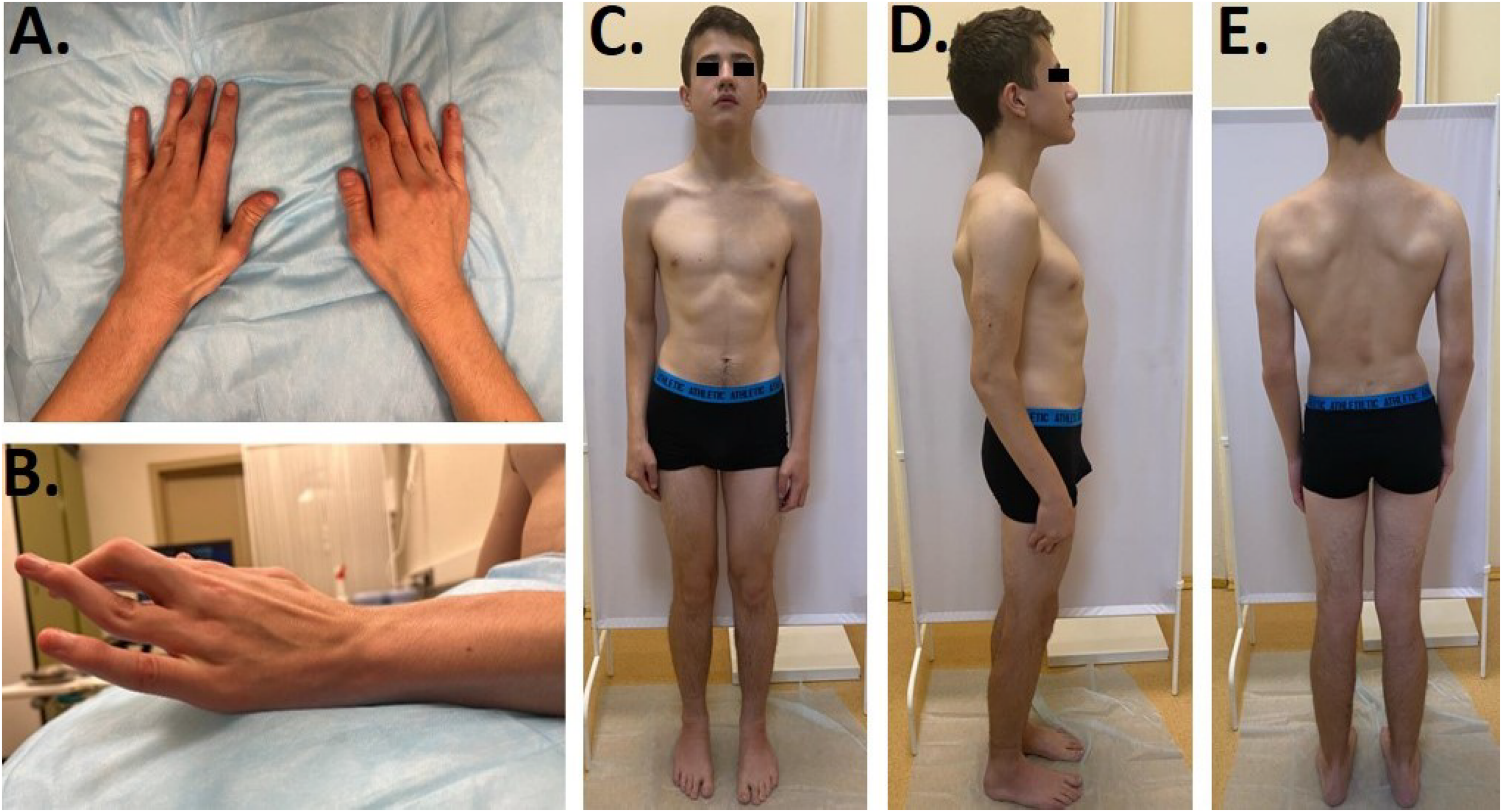
**(A**,**B)** Clinodactyly and abnormal finger morphology. **(C**–**E)** Patient's phenotype.

Furthermore, we noticed camptodactyly, deformation, and limited range of motion in the interphalangeal joints (Figures 1A,B), deformity of the chest, and asymmetric posture. Limited extension of the elbows (160°) was found, along with a limited range of motion of the wrists with flexion of −70° and extension of −80°, postoperative scars on the projections of the first and third fingers, “swan neck”-type deformations of the third and fourth fingers of the right hand and the fourth finger of the left hand, and flexion contracture of the third finger on the left hand. When walking, the patient rotated his lower extremities inward and limped on his left leg. There was an asymmetry of internal rotation (D > S) in his hip joints, a valgus deformity and shortening (1 cm) of his left lower limb, and planovalgus deformities of both feet (Figures 1C–E). According to the neurological examination, the patient had normal muscle tone and strength, hyporeflexia of the upper limbs, and normal tendon reflexes in his lower limbs. Surface and needle EMG did not show any differences.

**Figure 2:**
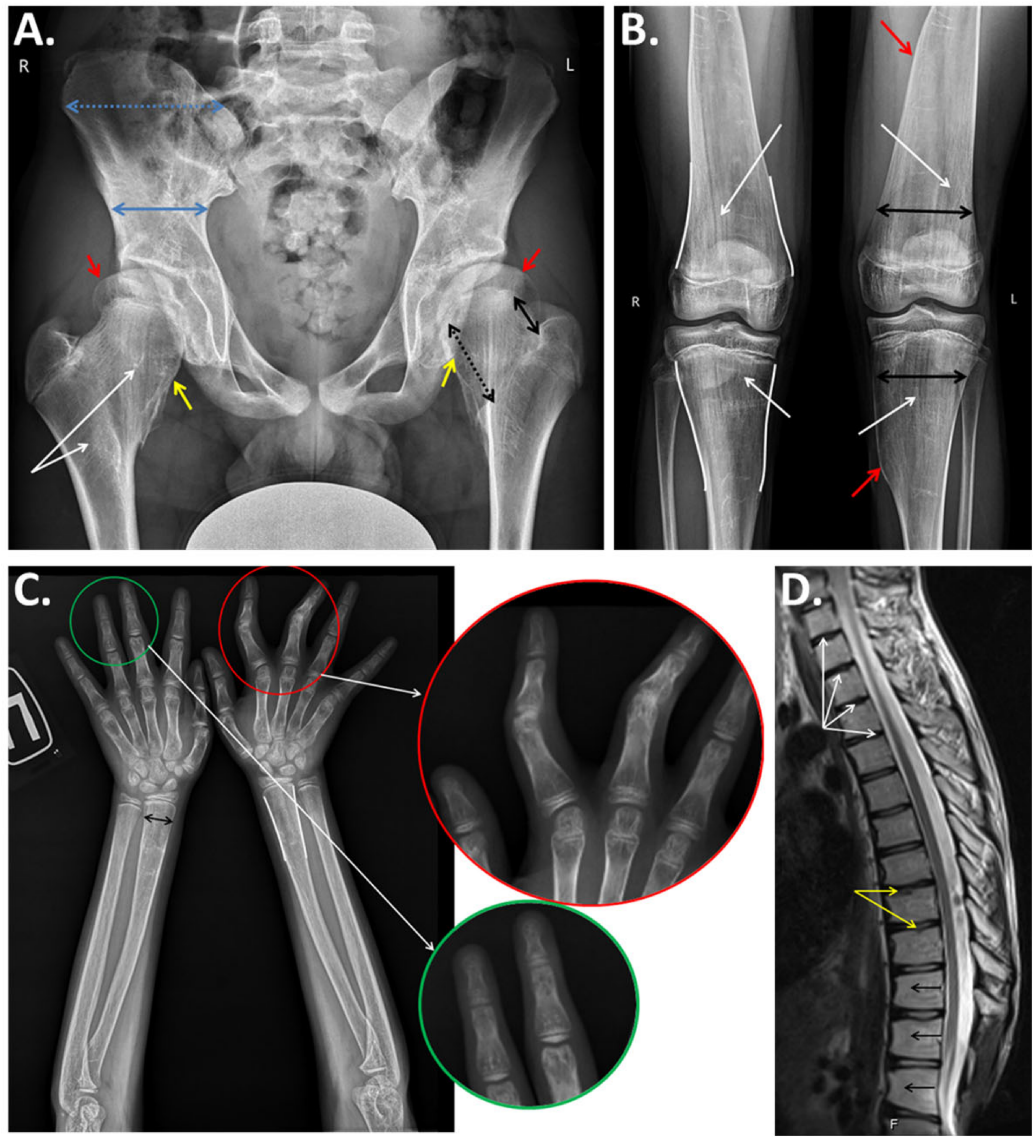
(**A**) Anteroposterior radiograph of the pelvis and hips of the proband demonstrates generalized derangement of the bone architecture with patchy dense and loose regions and striation of the metaphyses (white arrows); asymmetrical growth of the femoral neck with predominant growth of the medial part (black arrows), resulting in a widening with an exostosis-like medial prominence (yellow arrows) and the lateral extrusion of the femoral head (red arrows); and abnormal formation of the iliac bones with a relative widening of the isthmus and narrowing of the iliac wing (blue arrows). (**B**) Anteroposterior radiographs of the knee joints: abnormal bony structures with vertical striations of the metaphyseal areas (white arrows), undertubulation of the metaphyses, an “Erlenmeyer flask”-type widening of the metaphyses (white lines), and noticeable asymmetry of the “flask” with a medial exostosis-like prominence (red arrows). (**C**) Anteroposterior radiographs of the forearms and hands of the proband: “Erlenmeyer flask”-like deformities (white lines) and widening (black arrow) of the metaphyses of the radii, widening of the metacarpal bones, clinodactyly of the second and third digits with a narrowing of the interphalangeal joints (enlarged in the red circle), and cystic transformation of the metaphyses and epiphyses of the phalanges (enlarged in the green circle). (**D**) MRI (sagittal) of the thoracic spine: dehydrated intervertebral discs (white arrows), degenerative changes in the vertebral body endplates (yellow arrows), and anterior wedging of the vertebral bodies (black arrows).

Radiographic data (Figure 2) showed interesting findings, including a generalized disarrangement of the patient’s bony architecture with dense and loose zones, largely in the metaphyseal regions, with characteristic vertical striation of the metaphyses. An interesting and presumably characteristic feature was the asymmetric undertubulation of the metaphyses, resulting in the “misshapen Erlenmeyer flask” shapes of the distal radius, distal femur, and proximal tibia (Figures 2B,C). The unique result of the asymmetric undertubulation at the proximal femur resulted in the “exostosis-like” prominence of the medial part of the femoral neck which led to the preliminary hypothesis of multiple exostoses at the early diagnostic stage. As in typical exostoses of the proximal femur, this specific pattern of abnormal growth led to the lateralization and subluxation of the femoral head (Figure 2A). Interestingly, this asymmetric process also led to the deformity of the other segments with a secondary valgus knee deformity [the patient was operated on previously with eight-Plate™ (Orthofix, TX, USA)] and mild ulnar deviation of the wrist. A radiograph of the patient’s hands revealed the metaphyseal changes with secondary deformities of the fingers (Figure 2D).

Summarizing the radiographic data and comparing them with the previously published data, we noted that all of the aforementioned features were found and described before as separate symptoms, and this specific case gave us the unique chance to follow the evolution of these symptoms and propose some mechanisms underlying the general process of this condition within the bones and cartilaginous tissues.

### 3.2 Molecular findings

**Figure 3:**
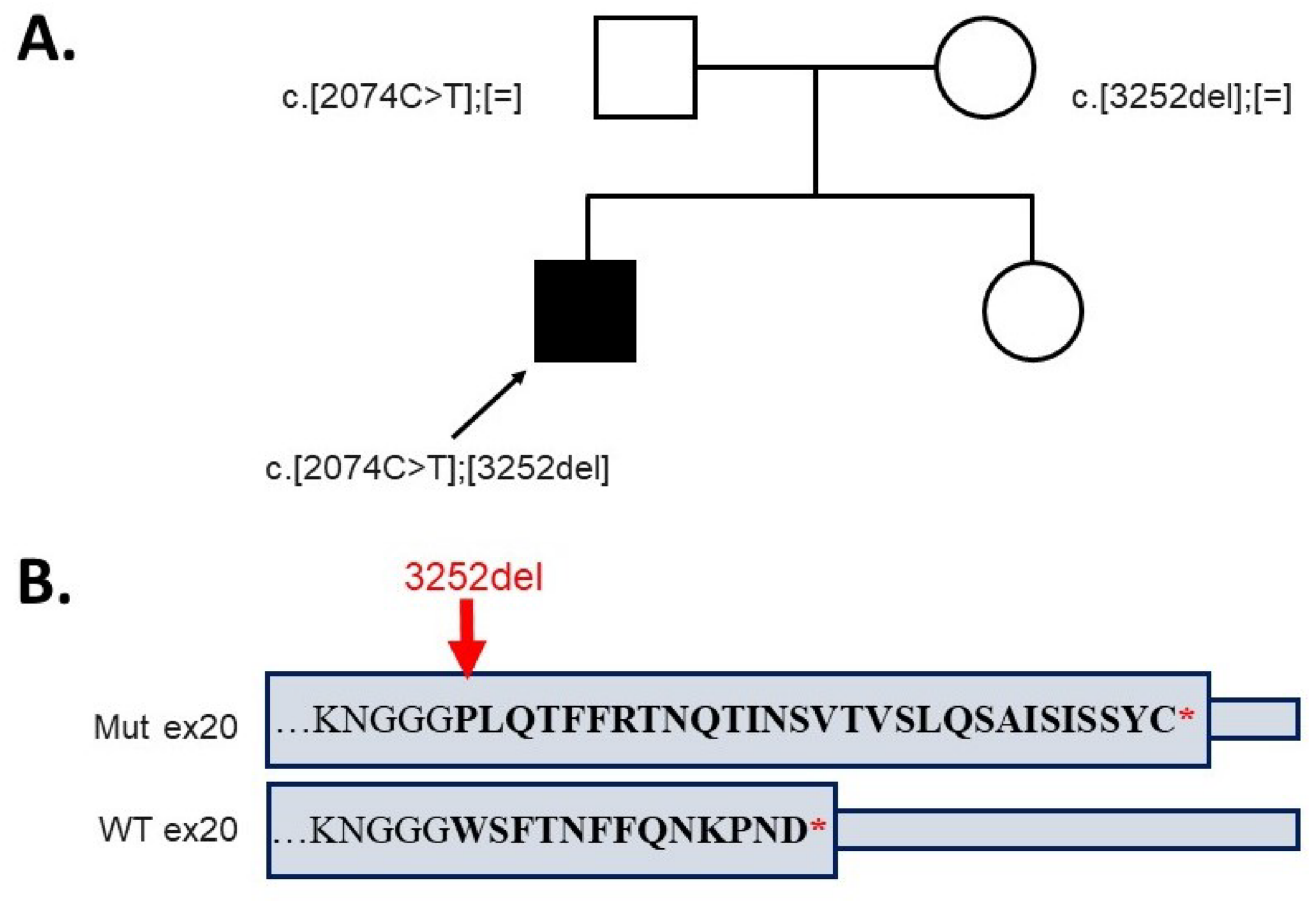
(**A**) The genotypes of the proband and his parents. (**B**) The alteration of the amino acid sequence in the last exon of the allele with variant c.3252del compared to the wild type.

Clinical exome sequencing revealed a previously described variant (10) in exon 15 of the *LIFR* gene, c.2074C>T, leading to a nonsense substitution, p.Arg692Ter, in the heterozygous state, which has been classified as pathogenic (PM2, PVS1, and PP5). Furthermore, a novel variant in the exon 20 of the *LIFR* gene, c.3252del, resulted in a frameshift mutation, p.Trp1085GlyfsTer31, in the heterozygous state, which has been classified as likely pathogenic (PM2, PM4, and PM3). The identified variants were confirmed to be in a compound heterozygous state in the proband using Sanger sequencing (the variant c.2074C>T was inherited from the father and c.3252del from the mother) (Figure 3A).

## 4 Discussion

Stüve–Wiedemann syndrome is a severe autosomal recessive bent bone dysplasia, which requires early diagnosis due to its high neonatal lethality and the potential for accompanying manifestations of autonomic dysfunction in children. The majority of SWS cases are associated with the *LIFR* gene.

Herein, we presented a patient with a late-onset form of SWS. His skeletal phenotype was mild compared to the other non-lethal cases of SWS described in the literature. The disease was not noticed until adolescence when his gait and posture abnormalities became significant. Currently, fewer than 10 patients who have survived into adolescence and beyond have been reported in the literature, usually with loss of function (LoF) variants in compound heterozygous or homozygous states (Supplementary Table S1) (4, 6–8, 11, 14, 17, 18). Progressive skeletal deformities in some cases have led to the loss of independent mobility by late childhood and necessitated early orthopedic surgery. In our patient, only camptodactyly and minor facial features were present at birth, thus his condition was assumed to be the arthrogryposis-like phenotype.

Observed clinical and radiological manifestations in the patient allowed us to confirm the diagnosis of atypical benign SWS. Autonomic dysregulation in this case was associated with early-life feeding challenges resulting in weight deficit, heat intolerance, and adolescent-onset episodes of prolonged sweating. Recurrent limb bone fractures between the ages of 5 and 11 years in addition to the gradual progression of a limitation of movement in the interphalangeal, radiocarpal, and elbow joints; a valgus deformity of the lower limbs; and gait disturbances caused us to suspect skeletal dysplasia. The radiographic examination indicated metaphyseal involvement initially indicative of metaphyseal dysplasia/Pyle disease. However, the patient did not have bent bone features, but rather a trabecular pattern of widened metaphyses, significant thickening of the femoral necks, and reduced epiphyseal heights with bone rarefaction.

The mild phenotype observed in our patient likely correlates with the presence of a novel heterozygous variant, c.3252del (p.Trp1085GlyfsTer31), located in the exon 20 of the *LIFR* gene, leading to a frameshift mutation. At the protein level, it alters 13 terminal amino acids with a further elongation of the C-terminus by 17 amino acids (Figure 3B). The elongated protein may be partly functional, explaining the extremely mild phenotype of Stüve–Wiedemann syndrome in our patient. Further functional studies are required to confirm the presumed molecular effect of the detected variant in the *LIFR* gene.

Thus, we presented a patient with an unusual phenotype of mild late-onset Stüve–Wiedemann syndrome. Exome sequencing revealed two variants in a compound heterozygous state in the *LIFR* gene: one was a previously described LoF variant, while the other was a novel C-terminal variant that altered the amino acid sequence in the cytoplasmic terminal domain, resulting in a milder phenotype. The patient lacked the classic features of bent bones and associated SWS complications at birth, had normal growth, and had orthopedic problems that manifested closer to adolescence. This case expands the known spectrum of variants in the *LIFR* gene and our knowledge of the variability of the non-lethal SWS phenotype, which will contribute to earlier detection, prediction of potential complications, and improvement of medical-genetic counseling.

## Data Availability

The datasets presented in this article are not readily available because of ethical and privacy restrictions. Requests to access the datasets should be directed to the corresponding author.
