# Supplementary material for: Case Report: New phenotype of late-onset Stüve–Wiedemann syndrome due to a C-terminal variant in the LIFR gene
Source: Front Pediatr. 2024 Oct 31;12:1442624. doi: 10.3389/fped.2024.1442624 (PMC11565277; doi:10.3389/fped.2024.1442624)
Supplement: Supplementary file 1 [file Table1.docx]

| Features | Case 1  Present | Case 2  Di Rocco, 2003 | Case 3  Reither M., 2006 | Case 4  Gaspar I. M., 2008 | Case 5  Jung С., 2010 | Case 6  Jung С., 2010 | Case 7  Melone MA, 2014 |
| --- | --- | --- | --- | --- | --- | --- | --- |
| **GENERAL** | | | | | | | |
| Age (years) | 15 | 12 | 16 | 12 | 14 | 12 | 33 |
| Ethnicity | Russia | Italy | Germany | Portuguese  Gypsy | France | France | Italy |
| Consanguinity | - | + | + | + | - | - | - |
| Gender | M | M | M | F | M | M | F |
| **MOLECULAR** | | | | | | | |
| *LIFR* variants | c.2074C>T (p.Arg692Ter)  c.3252del  (p.Trp1085GlyfsTer31) | ND | ND | c.167_170delTAA | c.875T>C  c.1438-4A>G | ND | c.2170C>G, (p.Pro724Ala) |
| Zygosity | Compound heterozygous | ND | ND | Homozygous | Compound heterozygous | ND | Izozygous |
| **CLINICAL** | | | | | | | |
| Midface hypoplasia | - | - | + | + | ND | ND | - |
| Short and wide nose | - | - | + | + | ND | ND | - |
| Pursed mouth /facial hypomimia | -/+ | +/+ | +/+ | +/+ | ND | ND | -/+ |
| Abnormality of the lips | + | + | + | + |  |  | - |
| Poor dentition/Loss of teeth/Dental decay | +/-/+ | -/-/+ | ND | +/+/+ | +/+/- | ND | ND |
| Feeding problems/ Need for tube feeding or gastrostomy | +/- | +/+ | +/+ | +/+ | +/ND | +/ND | +/+ |
| Respiratory distress/ Need for ventilation | -/- | +/- | +/+ | +/+ | +/ND | +/ND | +/- |
| Recurrent infections | - | - | + | + | ND | ND | ND |
| Motor delay | + | ND | + | + | + | + | + |
| Intellectual disability | - | - |  | - | - | - | - |
| Wheelchair dependent | - | ND | + | + | ND | ND | - |
| **DYSAUTONOMIA** | | | | | | | |
| Poor temperature regulation/paradoxical sweating | +/+ | +/+ | +/+ | +/+ | + | + | + |
| Reduced pain sensation | - | - | ND | + | - increased | - increased | ND |
| Absent corneal reflex | - | + | ND | + | + | ND | ND |
| Corneal injuries | - | + | + | + |  |  | ND |
| Smooth tongue/ Tongue ulceration | +/- | +/+ | ND | +/+ | +/ND | +/ND | ND |
| Hyporeflexia | + upper limbs | + | ND | + | - | - | ND |
| **SKELETAL** | | | | | | | |
| Short stature | - | + | + | + | + | + | - |
| Short bowed limbs | - | + | + | + | + | + | - |
| Enlarged joints | - | + | + | + | + | + | - |
| Contractures | + | + | + | + | + | + | + |
| Spine deformity, scoliosis | + | + | + | + | + | + | + |
| Camptodactyly | + | - | - | + | + | - |  |
| Talipes valgus/equinovarus | +/- | +/- | +/- | -/+ | + | + | -/+ |
| Fractures | + | - | + | + | + | + | - |
| **RADIOLOGICAL** | | | | | | | |
| Bowing and shortening of long bones | - | + | + | + | ND | ND | - |
| Dyaphiseal/metaphyseal undertubulation | -/+ | -/+ | -/+ | +/- | ND | ND | - |
| Abnormal trabecular pattern metaphyses | + | + | + | + | ND | ND | - |
| Wide metaphyses | + | + | + | + | ND | ND | - |
| Internal cortical thickening | + | + | + | + | ND | ND | - |
| Destruction of femoral heads | - | - | + | - | ND | ND | - |
| Osteoporosis/osteopenia | + | ND | + | + | ND | ND | - |

ND, non documented

Supplementary Table 1. Clinical and radiological features of long-term SWS survivors beyond 12 years of life
